# Are the anatomical, clinical, and ultrasound characteristics of thyroid nodules with Bethesda III or IV cytology and ACR TI-RADS 3, 4, or 5 able to refine the indications for molecular diagnostic tests?

**DOI:** 10.20945/2359-3997000000402

**Published:** 2021-09-29

**Authors:** Gustavo Cancela e Penna, Camila Teixeira Costa, Magda Carvalho Pires, Tarcizo Afonso Nunes

**Affiliations:** 1 Universidade Federal de Minas Gerais Faculdade de Medicina Departamento de Clínica Médica Belo Horizonte MG Brasil Departamento de Clínica Médica, Faculdade de Medicina, Universidade Federal de Minas Gerais, Belo Horizonte, MG, Brasil; 2 Universidade Federal de Minas Gerais Belo Horizonte MG Brasil Programa de Pós-Graduação em Ciências Aplicadas à Cirurgia e à Oftalmologia, Universidade Federal de Minas Gerais, Belo Horizonte, MG, Brasil; 3 Universidade Federal de Minas Gerais Departamento de Estatística Belo Horizonte MG Brasil Departamento de Estatística, Universidade Federal de Minas Gerais, Belo Horizonte, MG, Brasil

**Keywords:** Thyroid nodules, indeterminate nodules, indeterminate thyroid nodules, molecular tests, molecular diagnostic tests

## Abstract

**Objective:**

To analyze the association of clinical, anatomical, and ultrasound (US) characteristics of malignancies in Bethesda III or IV (III-B or IV-B) thyroid nodules.

**Subjects and methods:**

The association between malignancies and the following variables were analyzed: III-B or IV-B, age < 55 years and ≥ 55 years, sex, family history of thyroid cancer, history of irradiation, nodule size, and ACR TI-RADS classification in 62 participants who underwent thyroidectomy.

**Results:**

Of the 62 participants, 87.1% (54/62) were women, 74.2% were < 55 years old, 95.2% had no family history of thyroid cancer, 56.5% had nodules < 2 cm in size, 62.9% were IV-B, and 69.4% were ACR TI-RADS 4. Thirty-two patients had thyroid carcinoma, and 30 had benign histology. Among all factors associated with malignancy, only ACR TI-RADS 5 classification on US was found to be statistically significant (p = 0.014), while III-B with architectural atypia cytological classification was the only one significantly associated with benign status (p = 0.004).

**Conclusion:**

Only a high risk of malignancy as assessed using US was able to refine the indication for molecular tests in a group of patients with indeterminate nodules. We found 85% (53/62) of III-B or IV-B thyroid nodules would benefit from available molecular diagnostic tests.

## INTRODUCTION

The detection of thyroid nodules has increased considerably in recent decades, and most present with benign histology. The expansion of the use of ultrasound (US) for diagnosis and screening has led to the greater detection of nodules, causing a “diagnostic epidemic” ( 1 ). US can be used to estimate the probability of malignant nodule status and select whether to perform a fine-needle aspiration biopsy (FNAB) ( 2 - 6 ). FNABs of thyroid nodules accurately classify most nodules; however, the results remain undetermined in approximately 20% (10%-38%) when specific cytological characteristics necessary for definitive diagnosis are lacking ( 7 ). In this scenario, the growing number of biopsies, with the indeterminate cytological results that accompany the increased detection of thyroid nodules and cancers, is relevant ( 8 ).

Indeterminate cytology involves three Bethesda categories ( 9 ): Bethesda category III (III-B), which includes atypia of undetermined significance/follicular lesion of undetermined significance (AUS/FLUS); Bethesda category IV (IV-B), which includes follicular neoplasm/suspicious for follicular neoplasm or Hurthle cells (FN/SFN); and Bethesda category V (V-B), which is suggestive of malignancy. In a recent meta-analysis ( 10 ), thyroid malignancy corresponded to one-third of the resected nodules, with half of all resected nodules corresponding to indeterminate cytologies, and two-thirds of these cytologies representing benign nodules ( 11 ). In addition, approximately 25% of thyroid nodule FNABs are classified in the indeterminate Bethesda categories (III-B, IV-B, V-B), and more than 75% of III-B or IV-B cytology nodules have benign histology ( 3 , 10 ). Therefore, many of these nodules are referred for surgery due to the 10%-40% risk (category III-B or IV-B) of malignancy. This excess treatment exposes patients to short- and long-term surgical complications, in addition to permanent hormonal replacement with levothyroxine following total thyroidectomy and sometimes following lobectomy.

Currently, the most used US classifications for the evaluation of thyroid nodules are from the “American Thyroid Association Management Guidelines for Adult Patients with Thyroid Nodules and Differentiated Thyroid Cancer” (ATA 2015) ( 2 ) and “American College Radiology (ACR) Thyroid Imaging, Reporting and Data System (TI-RADS)” (ACR TI-RADS) ( 3 ), which are concordant in most thyroid nodules ( 12 ). The classification of ATA 2015 involves five categories: benign, very low risk, low risk, intermediate risk, and high risk, with the risk of malignancy being <1%, <3%, 5%-10%, 10%-20%, and >70%, respectively. The ACR TI-RADS classification is a system that uses scoring based on whether the nodule presents or does not present with malignant characteristics, and is classified from ACR TI-RADS 1 to ACR TI-RADS 5. The score ranges from 0 to 14 points, with higher scores representing a greater risk of malignancy for a given nodule. The characteristics involved in this classification include composition (solid, cystic, mixed), echogenicity (anechoic, hyperechoic, isoechoic, hypoechoic), shape (taller than wide or wider than tall), margins (well-defined, ill-defined, lobed, extrathyroid extension), and presence of echogenic foci (macrocalcifications, peripheral calcifications, punctiform echogenic foci). US characteristics influence the pretest value of the risk of malignancy when examining the cytology of a nodule, as shown by Rosario ( 13 ) in a prospective study; in the AUS/FLUS category, US analysis showed a sensitivity, specificity, positive predictive value (PPV), and negative predictive value (NPV) of 79.4%, 90.5%, 71%, and 93.75%, respectively, in predicting the malignancy of the nodules. Solid nodules that are markedly hypoechogenic or with microcalcifications, or that are hypoechogenic associated with another suspicious characteristic (irregular/microlobulated margins, anteroposterior diameter greater than transverse diameter, or central vascularization) are considered “suspect” ( 13 ).

According to the second edition of the Bethesda categorization published in 2017, the risk of malignancy is 10%-30% for III-B, 25%-40% for IV-B, and 50%-75% for V-B, disregarding non-invasive follicular thyroid neoplasm with papillary-like nuclear features (NIFTP), and 6%-18%, 10%-40%, and 45%-60%, respectively, when considering NIFTP histologies ( 9 ). There is heterogeneity within III-B, in which groups with nuclear atypia (NA) have a 2.6-fold risk (odds ratio [OR] = 3.63 in III-B; OR = 4.38 in IV-B) compared to groups with only architectural changes (AA) ( 1 , 14 ). Finally, V-B, generally considered indeterminate, is found in 2%-3% of all FNABs ( 7 , 11 , 14 ) and may present with a malignancy rate of 50%-75% ( 14 ) or 53%-97% according to the study ( 11 ). The focus of this study is III-B and IV-B, with each found in approximately 10% of all FNABs, since V-B already presents with a high risk of malignancy, which is sufficient surgical indication in most cases (high pre-test probability).

In this scenario, the use of molecular tests has gained importance ( 15 ), since it is necessary to use tools to assist in the preoperative diagnostic definition of thyroid nodules that are not highly suspected of being malignant or benign because of the characteristics available at this stage of the investigation (US, cytological, risk factors for thyroid carcinoma). Molecular diagnostic tests can be useful to avoid unnecessary surgeries in patients with thyroid nodules with III-B or IV-B cytology; however, their use is limited by their high cost. Zanocco and cols. ( 16 ) recently suggested that molecular tests are cost-effective in nodules with III-B or IV-B cytology with ACR TI-RADS 3 or 4 classification on US and cases with a pretest probability of benignity above 31% (some cases of ACR TI-RADS 2 with other risk factors and ACR TI-RADS 3 and 4), since in nodules with highly suspicious US (ACR TI-RADS 5), the tests showed benignity in only 8.3% of nodules, which means the tests are not cost-effective in these cases ( 17 , 18 ). ACR TI-RADS 2 and ACR TI-RADS 5 nodules already have a high pre-test probability for benignity and malignancy, respectively. Typically, the molecular test does not modify the treatment strategy, except in ACR TI-RADS 2 nodules with several other risk factors for malignancy (age, sex, size, previous irradiation history, positive family history, growth in follow-up period) or in ACR TI-RADS 5 nodules (<1.5 cm) without the presence of any of the other risk factors mentioned before.

Molecular tests, initially classified as “rule-in” and “rule-out”, were developed in recent years to improve the diagnostic accuracy of FNAB cytology ( 17 , 18 ). They consisted of small panels of genetic mutations that offered high PPV for cancer detection; however, they did not achieve sufficient NPV to reliably exclude malignancy in negative samples ( 19 , 20 ). Later, more advanced molecular tests were developed using gene expression technology, wider panels of mutational markers or combinations of different markers ( 21 - 25 ), and algorithms built with the aid of artificial intelligence, which offered greater sensitivity and improved NPV ( 26 , 27 ). Specifically, when the diagnostic test is designed to predict benign nodules and exclude malignancy, a high NPV is required. However, when predicting malignancy, a high PPV is required. According to Vargas-Salas and cols. ( 28 ), to consider a test to have a good ability to rule out malignancy, the test must have an NPV of at least 95%, which means that the residual risk of malignancy would be less than 5% for a negative result. This is close to the risk of malignancy for II-B cytology ( 28 ), and a minimum sensitivity above 86% is required to keep the NPV above 95% for a wide range of disease prevalences. There is no consensus on the minimum PPV necessary to consider a rule test adequate; however, a specificity rate above 87% would result in a PPV above 70% for a disease with a prevalence rate above 25% ( 29 ). The two tests available in Brazil have an NPV and sensitivity greater than 94% ( 30 - 32 ).

Therefore, the use of diagnostic molecular tests has gained feasibility to assist in the preoperative diagnostic definition, mainly of ACR TI-RADS 3 and 4 nodules with III-B and IV-B cytology. However, there are still doubts as to whether the clinical, cytological, and US characteristics associated with the risk of malignancy, whether alone or considered together, would be sufficient to dispense with the use of molecular diagnostic tests.

To answer these questions, this study evaluated whether anatomical and clinical data and the risk of malignancy based on US available in the preoperative period could contribute to a more selective indication for these tests in III-B or IV-B thyroid nodules. Risk factors for thyroid cancer were prospectively analyzed ( 33 - 35 ), including a positive family history of thyroid cancer, age, sex, and nodule size, in addition to Bethesda cytological and US ACR TI-RADS classifications, to determine which anatomical, clinical, and US characteristics, alone or considered together, would be able to predict the malignancy or benignity of III-B or IV-B nodules, in the preoperative period, to dispense with the use of molecular panels.

## SUBJECTS AND METHODS

In a cohort of 62 participants with III-B and IV-B cytology, who underwent thyroidectomy (total or partial) at the recommendation of the attending physician, with ACR TI-RADS 3 and 4 or ACR TI-RADS 5 on US, the association of malignancy with the following variables was analyzed: sex (female vs. male), age (<55 years; ≥55 years), family history of thyroid cancer, presence of thyroid cancer in first-degree relatives (yes vs. no), size of thyroid nodule (<2 cm vs. 2-4 cm vs. >4 cm), Bethesda classification (III-B only with AA, III-B with NA without cracks or pseudoinclusions, III-B with NA with cracks or pseudoinclusions, and IV-B), and ACR TI-RADS classification (ACR TI-RADS 3, 4, and 5). The variables are described in terms of absolute and relative frequencies. Logistic regression was used to assess factors associated with malignancy.

This prospective cohort study was approved by the Research Ethics Committee of the Federal University of Minas Gerais (CAAE number 17794719.9.0000.5149). All patients were informed about the study objectives and freely signed an informed consent form.

## RESULTS AND DISCUSSION

Sixty-two nodules were analyzed in 62 participants (one nodule in each participant), of whom 87.1% (54) were women, 74.2% ( 36 ) were < 55 years old, and 95.2% (59) had no family history of thyroid cancer. Among these, 56.5% ( 34 ) had nodules < 2 cm in size, 9.7% ( 6 ) had nodules > 4 cm in size, 62.9% were IV-B, and 69.4% were ACR TI-RADS 4. All nodules were resected; 32 were determined to be thyroid carcinoma and 30 were benign tumors ( Table 1 ).

**Table 1:** Anatomical, clinical, and ultrasound characteristics regarding the anatomopathological results of thyroid nodules

		Benign	Malignant	p-value
Sex				0.1006
	Female	24 (44%)	30 (56%)	
	Male	6 (75%)	2 (25%)	
Age				0.8809
	<55 years	22 (48%)	24 (52%)	
	≥55 years	8 (50%)	8 (50%)	
Family History				0.5888
	No	29 (49%)	30 (51%)	
	Yes	1 (33%)	2 (67%)	
Size				0.1708
	<2 cm	16 (46%)	19 (54%)	
	2-4 cm	9 (43%)	12 (57%)	
	>4 cm	5 (83%)	1 (17%)	
Bethesda				0.0040
	III-B with AA	3 (100%)	0 (0%)	
	III-B with NA (with cracks or pseudoinclusions)	1 (14%)	6 (86%)	
	III-B with NA (without cracks or pseudoinclusions)	3 (23%)	10 (77%)	
	IV-B	23 (59%)	16 (41%)	
ACR TI-RADS				0.01398
	ACR TI-RADS 3	7 (54%)	6 (46%)	
	ACR TI-RADS 4	23 (53%)	20 (47%)	
	ACR TI-RADS 5	0 (0%)	6 (100%)	
ATA 2015				0.01574
	Low	7 (41%)	10 (59%)	
	Intermediate	23 (57%)	17 (42%)	
High		0 (0%)	5 (100%)	

The average age of patients was 45.27 years, and the majority of patients were female; however, there was no statistical significance regarding carcinomas (p = 0.1006, Table 1 ). There was also no statistical significance regarding family history of thyroid cancer in first-degree relatives or size of the nodules divided into < 2 cm, 2-4 cm, and > 4 cm in size. However, it is important to highlight a reduction in the p-value (0.06172) when simplifying the analysis to nodules ≤ 4 cm and > 4 cm, which indicates a tendency towards greater risk of malignancy in larger nodules, as suggested by some studies ( 35 ) ( Table 2 ). The III-B classification was divided into three subtypes: III-B with AA, III-B with NA other than cracks or pseudoinclusions, and III-B with NA with cracks or pseudoinclusions. These subtypes were described in the latest edition of the Bethesda classification published in 2017 as nuclear atypia due to the presence of enlarged and prominent nuclei with pale chromatin, in addition to rare pseudoinclusions in the AUS/FLUS ( 11 ) classification and that in these groups with nuclear atypia there was a 2.6-fold risk in relation to the group that presented with architectural changes only ( 1 , 14 ). The results obtained were 100% benignity in nodules where only architectural changes were found. In nodules with nuclear alterations, other than cracks or pseudoinclusions, there was a predominance of malignant cases (77% malignant vs. 23% benign) and in nodules that were III-B with cracks and pseudoinclusions, the presence of malignancy was even greater (86% malignant vs. 14% benign), as shown in Table 1 . Again, there was a reduction in the p-value (0.0040 to 0.0014), suggesting a greater statistical association with malignancy when simplifying the analysis to III-B with nuclear atypia vs. IV-B ( Table 2 ). The high rate of malignancy found (32/62) in the III-B and IV-B categories (51.6% vs. 20%-25%, which would be the expected malignancy rate for these two groups together), is justified by the careful surgical indication for all patients (ACR TI-RADS 5 and III-B and IV-B with some risk factors for malignancy, such as suggestive US characteristics). Regarding the high rate of malignancy in category III-B with NA (around 80%), the result was surprising and can be justified by the absence of well-defined criteria for this category. According to the latest Bethesda classification ( 9 ), the diagnosis of atypia of undetermined significance is reserved for samples containing cells (follicular, lymphoid, or others) with architectural atypia and/or atypia that are not sufficient to be classified as a suspected follicular neoplasia, suspected to be malignant, or malignant, reinforcing that atypia that are more accentuated than can be convincingly attributed to benign cytologies. The criteria for defining III-B are variable: cytological atypia, architectural atypia or both, presence of Hurthle cells, atypia not specified in another category, atypia with lymphoid cells, and lymphoma being ruled out. This broad definition allows the subjectivity of the examiner (depending on their experience and experience of the service in which they find themself) to be relevant in determining this classification.

**Table 2:** Subanalyses of anatomical, clinical, and ultrasound characteristics regarding the anatomopathological results of thyroid nodules

		Benign	Malignant	p-value
Size				0.06172
	≤4 cm	25 (44.6%)	31 (55.4%)	
	>4 cm	5 (83%)	1 (17%)	
Bethesda				0.001454
	III-B with AA	3 (100%)	0 (0%)	
	III-B with NA	4 (20%)	16 (80%)	
	IV-B	23 (59%)	16 (41%)	
ACR TI-RADS				0.003476
	ACR TI-RADS 3/4	30 (53.6%)	26 (46.4%)	
	ACR TI-RADS 5	0 (0%)	6 (100%)	
2015 ATA patterns				0.008038
	Low/Intermediate	30 (52.6%)	27 (47.4%)	
	High	0 (0%)	5 (100%)	

Although all cases in this study were examined by pathologists who perform a high volume of thyroid exams in a service where doubtful cases are reviewed by peers before making the diagnosis, we propose that a portion of the nodules classified as category III-B with NA are actually category V-B, and this is a risk that exists and has already been described in the literature ( 13 , 37 - 41 ). In the second step, it is possible to review these category III-B cases blindly using another reference service to assess the agreement and discuss the possible pitfalls of III-B classification. The rate of malignancy in IV-B patients (41% malignant) was slightly higher than that reported in the literature (15%-30%). The selective indication for surgery in IV-B cases at higher risk (when there are clinical, cytological, and US risk factors) justifies this finding.

Finally, in the US analysis, nodules classified as ACR TI-RADS 5 and high risk according to the ATA 2015 classification, presented with a 100% malignancy rate on histology. There was a small difference in the classification of low-risk and intermediate-risk ATA 2015 nodules compared to ACR TI-RADS 3 and ACR TI-RADS 4 ( Table 1 ). However, when combining “low risk/intermediate risk” and “ACR TI-RADS 3/ACR TI-RADS 4” in one category, the difference in the classification of the nodules was minimal, in addition to supporting the association between malignancy and ACR TI-RADS 5 and high-risk ATA 2015 (p = 0.003475 and 0.008038, respectively), as predicted in the literature ( 2 , 3 ) ( Table 2 ).

To assess risk factors for thyroid cancer and its prognosis, nomograms have already been described to validate their clinical use ( 42 - 44 ). However, the predictive power of these risk factors has not been compared with the predictive power of molecular tests to the point of dispensing with the indication of molecular tests. The agreement between the ACR TI-RADS and ATA 2015 classification was assessed using the kappa index, which had a value of 0.848 ( Table 3 ), showing substantial agreement. Similar to the analysis of this study, researchers at the Clinical Hospital of the Federal University of Paraná (UFPR) in Brazil ( 36 ) noticed the less aggressive behavior of some nodules classified as ACR TI-RADS 4 (TR4). Furthermore, the authors divided this category into TR4a (4 points), TR4b (5 points), and TR4c (6 points), where TR4a was equivalent to intermediate risk in ATA 2015 (with intermediate risk equivalent to low risk in the study). This study demonstrated a high NPV for III-B associated with a “favorable” US (TR2, TR3, TR4a/ATA 2015, very low risk to intermediate risk), similar to those presented in the molecular tests. The characteristics that demonstrated statistical significance were nodule size, markedly hypoechoic, higher than wide, irregular margins, and presence of microcalcifications, with no significant difference between age and sex, as shown in this study.

**Table 3:** Index of agreement between the ACR TI-RADS and ATA 2015 ultrasound classifications

ATA	ACR TI-RADS		
	3	4	5
Low	13	4	0
Intermediate	0	39	1
High	0	0	5
		kappa = 0.848	p = 0.000

In 2013, two studies evaluated factors indicative of worse prognosis related to thyroid cancer and death ( 40 , 41 ). In the first study, the probability of death at 5 and 10 years increased with advancing age, male sex, lesion size, and radiotherapy ( 40 ). The second study corroborated that the predictors of worse prognosis are age at diagnosis, male sex, TNM status, histology, presence of distant metastasis, post-treatment macroscopic residue, and lymph node involvement ( 41 ). In 2015, Lang and cols. developed a simpler nomogram to contribute to the individual assessment of patients with thyroid cancer instead of staging-based approaches that work better in population analyses than individual ones ( 42 ). The nomogram proposed the use of age, size, presence or absence of multifocality, lymph node involvement, and distant metastasis, and developed a numerical score, with values < 28 having high NPV, indicating a 99% chance of not dying in the next 10 years due to thyroid cancer. The study also indicated that the performance of molecular tests may contribute to better accuracy of the nomogram ( 42 ). The variables age and size were also included in our study, but did not contribute significantly to distinguishing between benign and malignant lesions.

A limitation of this study is the small number of patients, which may explain the lack of association of known risk factors for thyroid cancer related to the malignancy of the lesions studied (such as age, sex, family history, and previous exposure to radiation). Nevertheless, a significant statistical association between III-B cytology was found, with an emphasis on nuclear changes (which were more evident when these nuclear changes were cracks and/or pseudoinclusions), IV-B, and US with a high risk of malignancy, similar to analyses found in the literature ( 16 ).

We concluded that, despite the small number of participants, 85% (53/62) of thyroid nodules were classified as III-B (in this study, only those with NA) or IV-B, and in those that are also ACR TI-RADS 3 or ACR TI-RADS 4, molecular tests may provide benefits when determining the indication for surgery, given the inability of most analyzed anatomical and clinical characteristics, whether considered alone or together, to predict malignancy or benignity with statistical significance. The exception would be already high-risk nodules on US, ACR TI-RADS 5 (in this study, they were all malignant histologically) and, if confirmed in new studies, ACR TI-RADS 3 and ACR TI-RADS 4 III-B nodules with AA (in this study, there were three and all were benign). Therefore, regardless of the importance of using molecular tests, when suitably indicated (i.e., nodules that are category III-B or category IV-B and ACR TI-RADS 3 or ACR TI-RADS 4), we must always remember to individualize each approach, considering the high cost of these tests. We should also continually analyze categories III-B and IV-B before deciding on whether a molecular test is indicated and consider factors such as US characteristics (ACR TI-RADS 2 and ACR TI-RADS 5, usually presenting with a high pretest probability for benignity and malignancy, respectively, dispensing with the need for molecular testing), positive family history (three or more first-degree relatives affected by thyroid cancer), sex (increased risk of malignancy in men), nodules showing significant growth on serial US (if performed and available), history of previous head and neck or whole body irradiation, and cytology (III-B with NA or IV-B). Before using the molecular panel, it is necessary to evaluate the possibility of revising the slide and/or the new FNAB in category III-B. Only after analyzing all clinical, cytological, and US characteristics should whether molecular tests can assist in therapeutic decisions be considered. Finally, all the factors analyzed in this study allowed us to reach the conclusions already mentioned regarding the benefits of molecular tests in ACR TI-RADS 3 or ACR TI-RADS 4 and III-B or IV-B.
